# A Visual Cortex-Inspired Imaging-Sensor Architecture and Its Application in Real-Time Processing

**DOI:** 10.3390/s18072116

**Published:** 2018-07-02

**Authors:** Hui Wei, Luping Wang

**Affiliations:** 1 Laboratory of Cognitive Model and Algorithm, Department of Computer Science, Fudan University, No. 825 Zhangheng Road, Shanghai 201203, China; 15110240007@fudan.edu.cn; 2 Shanghai Key Laboratory of Data Science, No. 220 Handan Road, Shanghai 200433, China

**Keywords:** visual cortex-inspired architecture, constraint propagation, imaging sensor

## Abstract

For robots equipped with an advanced computer vision-based system, object recognition has stringent real-time requirements. When the environment becomes complicated and keeps changing, existing works (e.g., template-matching strategy and machine-learning strategy) are computationally expensive, compromising object recognition performance and even stability. In order to detect objects accurately, it is necessary to build an efficient imaging sensor architecture as the neural architecture. Inspired by the neural mechanism of primary visual cortex, this paper presents an efficient three-layer architecture and proposes an approach of constraint propagation examination to efficiently extract and process information (linear contour). Through applying this architecture in the preprocessing phase to extract lines, the running time of object detection is decreased dramatically because not only are all lines represented as very simple vectors, but also the number of lines is very limited. In terms of the second measure of improving efficiency, we apply a shape-based recognition method because it does not need any high-dimensional feature descriptor, long-term training, or time-expensive preprocessing. The final results perform well. It is proved that detection performance is good. The brain is the result of natural optimization, so we conclude that a visual cortex-inspired imaging sensor architecture can greatly improve the efficiency of information processing.

## 1. Introduction

In automotive robots, advanced perception-based systems (e.g., computer vision-based systems), raise a number of timing- and robustness-related issues. Some of these issues are related to the inefficiency introduced by the architecture and algorithm implementation on a given hardware platform. Especially in object recognition, existing solutions are computationally expensive, and descriptors based on machine learning are of high dimension. In particular, the problem addressed here is that due to the real-time requirements, the architecture and algorithm should not only be robust to environmental change, but should also be implemented efficiently in the quite complicated background.

Given the importance of object recognition to most computer vision-based systems, shape-based recognition is an active research area because shape is a steady and invariant cue for object recognition. In general, geometric models (shape-based) provide much more robust and useful information than photometric or other features. However, these models are prevented from being extensively used because of the inefficient architecture (i.e., the neural architecture).

In this paper, we build a visual cortex-inspired imaging sensor architecture and a method of constraint propagation examination to efficiently extract information. Using the imaging sensor with the proposed architecture and the aforementioned constraint propagation, target objects that mostly satisfy the geometric constraints of the shape-based model can be efficiently detected. The implementation of the proposed technique allows rapid application to the detection of an object in a quite-complicated environment. Finally, the location, scale, and orientation of an object can be estimated via the verification.

The remainder of this paper is organized as follows: Section 2 reviews the related work. Next, Section 3 describes the bio-inspired line detection architecture to efficiently detect lines. Then, Section 4 extends the efficient approach of line-based constraint propagation examination for object recognition. The experimental results of the proposed technique are shown in Section 5. Finally, a conclusion is given in Section 6.

## 2. Related Work

**Traditional object detection algorithms are inefficient.** In existing works, impressive theoretical progress has been made in shape-based object recognition [1,2,3,4,5,6,7,8,9,10,11,12,13,14,15,16,17,18]. There are classical features (HOG [19] and SIFT [20]), influential shape descriptor (shape context) [1], and the improved descriptors of greater complexity [5,6,7,21], but most of them can hardly be used because of their complicated computation that would compromise the real-time performance and even stability in hardware. Other methods such as the hierarchical shape matching method [8], unsupervised learning algorithm [22], and the fan model [23] are still incapable of meeting the requirements of efficiency for a computer vision-based system in robots.

**Improvement from a bio-inspired perceptive.** The human vision system is far superior in its efficient performance of objection detection to any current machine visual system [24,25]. Computational models for object recognition benefit from a biological foundation. Since Hubel presented a neural model on a receptive field [26], orientation features have attracted great attention from many researchers. Serre et al. [27] proposed the popular HMAX model in object recognition based on the orientation feature. Recently, Wei et al. [28,29] introduced the novel computational model of orientation detection simulating the mechanism of simple cells in the V1 area. Tomaso et al. [30] proposed the visual path in the primary visual cortex and promoted the building of more improved models that obey the rules of the visual cortex. There have also been some other methods, such as the combination of contour fragments [31], partial shape matching [32], optimal solution [33], and detection based on different models [34].

**Demands on vision computation.** The traditional methods of image processing require computational 10–1000 Gop/s, but the general microprocessing speeds (1–5 Gop/s) are directly related to the number of transistors on a chip, resulting in the inefficiency of image processing. Simulating the visual cortex with hardware is a very prosperous field.

However, the image analysis tasks that they focused on were at the signal processing level. Despite these extensive efforts, the precision and efficiency still fall short of the biological neural vision system. It is imperative to build an efficient imaging sensor architecture and a corresponding object detection method to process sensor information at human-level performance.

## 3. A Bio-Inspired Line Detection Architecture

**Figure 1:**
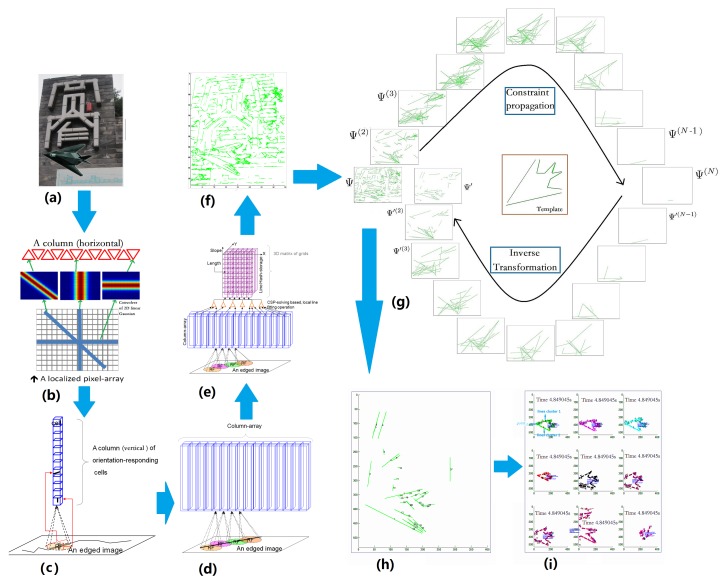
Architecture. (**a**) is the input image; (**b**) shows a basic functional unit. The slopes of linear stimulus for which these cells are responsible are different and exclusive. The response value of a cell is determined by its sensitive linear stimulus length and position, which can be implemented by a real-time convolver of a 2D linear Gaussian function. (**c**) A column of orientation-responding cells. A primary visual cortex-inspired column is composed of dozens of orientation-sensitive cells. They share a common receptive field on an image, but each cell is in charge of a specific and exclusive linear stimulus occurring in the receptive field (RF). (**d**) Column-arrays. A long line might pass through multiple RFs. Perceiving it can be seen as a fitting operation, subjected to multiple constraints provided by those RFs. A number of columns can be orderly arranged to form an array. The receptive fields of these columns might be partially overlapped. This array processes the image in a compound receptive field of all columns. (**e**) The architecture. At the top of column array, there is a 3D matrix of grids. Each grid is a register for storing a line. X and Y index the line’s center position, and the vertical position of the grid indexes the line’s slope, and the value stored in a grid is the line’s length. (**f**) An example of line extraction by the proposed architecture. (**g**) The process of constraint propagation. (**h**) Lines resulting from the constraint propagation. (**i**) The object estimation by verification. The location, scale, and orientation of the object can be verified through searching path. The top nine paths satisfying the geometric constraints of Γ were found. Each path represents an estimation of the object. It is obvious that our method not only efficiently detected the object but also estimated the location, scale, and orientation of the object.

The reason why our human visual system can process stimuli rapidly is that our brain is a highly optimized architecture. Compared with other sensory modes, the neural mechanism of vision has been studied relatively deeply. This benefited us while designing a bio-inspired architecture for image processing (Figure 1). Here we mainly refer the discovery of orientation columns in the primary visual cortex. Simply said, neuroscience proved that there are many vertical columns distributed in the visual cortex, and each of them is regarded as the basic functional unit for continuous orientation detection. That is, any linear stimulus with a slope value must be responded to exclusively by one of the cells belonging to a column, as long as this linear stimulus occurs in a small area (i.e., receptive field or RF) that this column is responsible for.

As shown in Figure 1b, a basic function alunit is composed of a limited number of orientation-sensitive cells. The slopes of the linear stimulus that these cells are responsible for are different and exclusive. The response value of a cell is determined by its sensitive linear stimulus length and position, which can be implemented by a real-time convolver of a 2D linear Gaussian function in which a better noise suppression with minimum edge blurring can be achieved.

A primary visual cortex-inspired column is composed of dozens of orientation-sensitive cells, as shown in Figure 1c. They share a common receptive field on an edge image (edges detected canny), but each cell is in charge of a specific and exclusive linear stimulus occurring in the receptive field.

A number of columns can be orderly arranged to form an array as shown in Figure 1d. The receptive fields of these columns might be partially overlapped. This array processes the image in a compound receptive field of all columns. The arrangement of receptive fields were introduced in [35].

Each cluster is in charge of a small area of the image (called receptive field, RF), and detects linear stimulus occurring in this RF. A long linear stimulus might pass through multiple receptive fields, activating dozens of cells. Supposing that a long linear stimulus has passed through *N* basic units field, the orientation and strength of the activated cell in each unit is $s_{i}$, $\sigma_{i}$, respectively. Then, this long linear stimulus can be fitted as the following equation:

(1) $$\left\{\begin{array}{c} x=x_{c}+tcos\alpha ,\\ y=y_{c}+tsin\alpha , \end{array}\right.$$

where the above line equation should satisfy the conditions as follows:

(2) $$arg_{x_{c},y_{c},\alpha }=Min\left(\sum_{i=1}^{N}{\left(tan\alpha -s_{i}\right)}^{2}\right),$$

(3) $$arg_{x_{c},y_{c},\alpha }=Max\left(\prod_{i=1}^{N}\frac{|length_{i}|}{Length_{i}}\right).$$

Here, $Length_{i}$ is the length of the template, and $length_{i}$ represents the projection of *i* linear stimulus to $Length_{i}$.

At the top of the column array shown in Figure 1e, there is a 3D matrix of grids. Each grid is a register for storing a line. X and Y index the line’s center position, the vertical position of the grid indexes the line’s slope, and the value stored in a grid is the line’s length.

The output of fitting would be transformed into parameter hash and stored. The range of columns that each fitting unit is responsible for can be determined by a competitive learning strategy. This arrangement of connections was determined by off-line training on many long linear stimuli.

**Figure 2:**
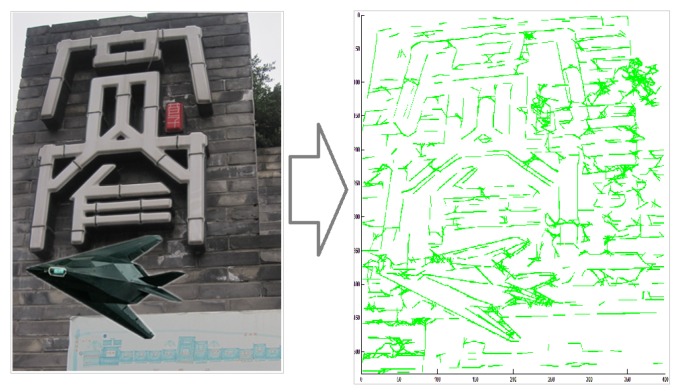
This shows an example of line extraction by the proposed architecture. From an image with complicated background in the **left** figure, it is possible to efficiently extract a number of lines as shown in the **right** figure.

By the proposed architecture it is possible to efficiently detect linear contour information (shown in Figure 2) as the neural architecture.

**Figure 3:**
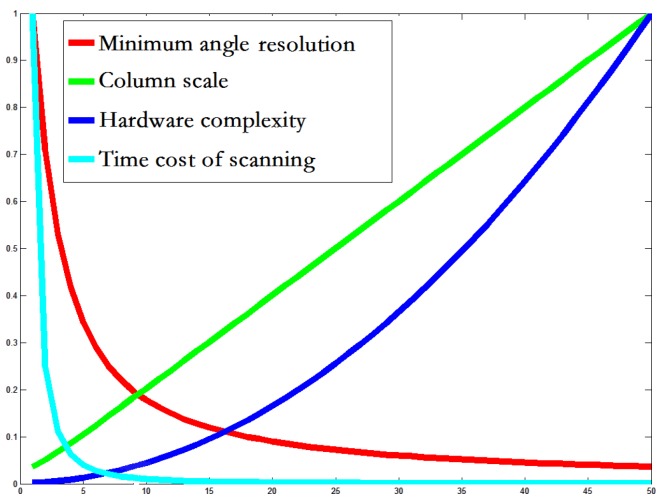
The relations to the complexity of logical RF in the architecture. The *x*-axis is the RF size of a column modular and the *y*-axis is normalized to adapt four different values: minimum angle resolution, RF size of a column, the total hardware complexity, and the time cost of scanning. Based on these four curves, we can find one or several balance points, at which the performance is not optimal but its corresponding cost is relatively low in the proposed architecture.

Firstly, the RF size of a column modular would inevitably affect the whole system’s complexity, orientation-perceiving resolution, and the time cost for an array scanning image. If the RF size is small, then the number of connections between cells and the RF will be small, resulting in a low structural complexity which would ease the layout of the hardware in a limited area. In addition, if the RF size is small, then the number of ideal short lines occurring in this 2D pixel matrix will be small, and then on one hand this would reduce the number of cells, and on the other hand this would increase the interval of two neighboring slopes (i.e., the angle-perceiving resolution would be decreased). On the contrary, with a large column modular RF, its angle-perceiving resolution would be be improved. At the same time, a large-scale RF would increase the number of cells and connections (i.e., high structural complexity). Secondly, the efficiency of image-scanning would be affected by the size of the RF and whole array. Obviously, compared with a small array, a large one must need less window-shifting and have a shorter moving distance when that array is searching an image. Thus, the time cost of scanning would be decreased. Of course, a large array needs higher hardware cost. Thirdly, a long line always needs multiple fitting operations because one scan from the array cannot cover it completely. So, a large array is advantageous because it can decrease the fitting time. However, a large array pays a greater complexity cost when connecting columns in a longer band-area. According to the aforementioned reasons, when designing our architecture we should consider the structural complexity, the detection performance, and the time cost. Here we conducted a quantitative experiment to analyze which sizes are more rational for the RF and for the array. Supposing that the shape of the RF is rasterized, the following can be approximately found:

(4) $$\begin{array}{c} f_{1}\left(x\right)=∥arctan\left(2/x\right)∥, \end{array}$$

(5) $$\begin{array}{c} f_{2}\left(x\right)=∥\varphi \pi /arctan\left(2/x\right)∥, \end{array}$$

(6) $$\begin{array}{c} f_{3}\left(x\right)=∥\varphi \pi /arctan\left(2/x\right)∥+∥x\varphi \pi /arctan\left(2/x\right)+\varphi^{2}/3∥, \end{array}$$

(7) $$\begin{array}{c} f_{4}\left(x\right)=∥4S/\left(3\varphi x^{2}\right)∥, \end{array}$$

where *x*, φ represent the scale of the basic logical RF and column array, respectively. *S* is the image size. $f_{1}$ is the minimum resolution angle. A smaller minimum resolution angle represents a higher angle resolution and precision of orientation-sensitive cells. $f_{2}$ means the number of cells in one column, and $f_{3}$ represents hardware complexity, including the number of cells and connections between cells and columns. $f_{4}$ means the time cost for scanning an image. In Figure 3, φ=20, S=800×640, the performance measures are normalized.

## 4. Line-Based Constraint Propagation Examination

Through the proposed architecture, the detected linear contour information were stored in a 3D matrix of grids, including the line’s center position, slope, and length. In order to efficiently detect the object, a constraint propagation approach was designed. By efficiently merging pair lines that satisfy the geometric constraint, it is possible to efficiently detect target objects that mostly satisfy the geometric constraints of the given shape-based model.

### 4.1. Constraint Propagation

**Figure 4:**
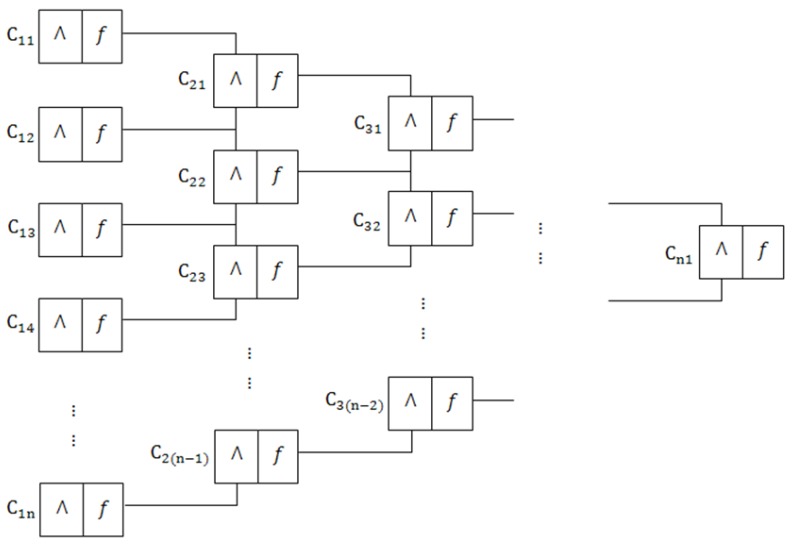
The process of constraint propagation. In the highest layer, $V_{(n)1}\left(f^{n}\right)=T$ means that one line in this layer represents a combination of basic lines that might satisfy the geometric constraints in $C_{11}$∼$C_{1(n)}$. $C_{1n}$ means the *n* constraint in the first layer. Λ means two lines satisfy the geometric constraint. *f* means two lines that satisfy the constraint are merged into a new line. By constraint propagation, it is possible to efficiently detect lines that satisfy the first layer constraints $C_{11}$∼$C_{1(n)}$.

As shown in Figure 4, supposing that there is a geometric constraint *C*, the process of satisfying *C* between line *i* and line *j* can be expressed as follows:

(8) $$C\left(i,j\right)=\left\{\begin{array}{c} TRUE(T),\\ FALSE(F), \end{array}\right.$$

where TRUE means that line *i* and line *j* satisfy the geometric constraint *C*. Then, the process of merging two lines into one line can be considered as a function as follows:

(9) $$V\left(z\right)=V\left(f\left(i,j\right)\right)=\left\{\begin{array}{cc} T & \;\;\;if\;\;C(i,j)=T,\\ F & \;\;\;if\;\;C(i,j)=F, \end{array}\right.$$

where *z* represents the new line merged by the line *i* and line *j*, and V(z)=T represents that the new merged line *z* is legal.

As shown in Figure 4, the process of constraint propagation can be proved as follows:

(10) $$\begin{array}{c} \begin{array}{c} V_{(n)1}\left(f^{n}\right)=T\\ \Rightarrow C_{(n)1}\left(f^{n-1},f^{n-1}\right)=T\\ \Rightarrow \neg (C_{(n)1}\left(f^{n-1},f^{n-1}\right)=F\\ \Rightarrow \neg V_{(n-1)1}\left(f^{n-1}\right)⋁\neg V_{(n-1)2}\left(f^{n-1}\right)=F\\ \Rightarrow \neg V_{(n-1)1}\left(f^{n-1}\right)=F⋀\neg V_{(n-1)2}\left(f^{n-1}\right)=F\\ \Rightarrow V_{(n-1)1}\left(f^{n-1}\right)=T⋀V_{(n-1)2}\left(f^{n-1}\right)=T\\ \Rightarrow C_{(n-1)1}\left(f^{n-2},f^{n-2}\right)=T⋀C_{(n-1)2}\left(f^{n-2},f^{n-2}\right)=T\\ \ldots \ldots \\ \Rightarrow C_{21}\left(f^{1},f^{1}\right)=T⋀\ldots ⋀C_{2(n-1)}\left(f^{1},f^{1}\right)=T\\ \Rightarrow C_{11}\left(f^{0},f^{0}\right)=T⋀\ldots ⋀C_{1(n)}\left(f^{0},f^{0}\right)=T\\ \Rightarrow C_{11}\left(f^{0},f^{0}\right)⋀C_{12}\left(f^{0},f^{0}\right)\ldots ⋀C_{1(n)}\left(f^{0},f^{0}\right)=T \end{array} \end{array}$$

Therefore, the demonstrated proposition means that it is proved to extract the basic lines by selecting the final line $V_{(n)1}\left(f^{n}\right)$ generated by performing the process of constraint propagation above. By constraint propagation, it is possible to efficiently detect lines that satisfy the first layer constraints $C_{11}$∼$C_{1(n)}$.

### 4.2. Constraint Propagation for Line Extraction

For a given shape-based template consisting of *N* lines,

(11) $$\begin{array}{c} {Ł}_{i}=[p_{i},l_{i},k_{i}]\;\;\;\;i\in N. \end{array}$$

Here, *N* is the number of straight lines in the image, and $p_{i},l_{i},$ and $k_{i}$ are the midpoint, length, and slope of line *i*, respectively. $\theta_{ij}$ is the angle of line *i* and line *j*, and

(12) $$\begin{array}{cc} \theta_{ij}=arctan\left|\frac{k_{i}-k_{j}}{1+k_{i}k_{j}}\right| & \;i\in N,j\in N, \end{array}$$

(13) $$\begin{array}{cc} \gamma_{ij}=l_{j}/l_{i} & \;i\in N,j\in N, \end{array}$$

(14) $$\begin{array}{cc} \zeta_{ij}=distance\left(p_{i},p_{j}\right)/L_{i} & \;i\in N,j\in N, \end{array}$$

where $\gamma_{ij}$ is the ratio of length and $\zeta_{ij}$ represents the ratio of distance to length. $distance(p_{i},p_{j})$ is the distance between point $p_{i}$ and $p_{j}$. For *N* lines, the geometric constraints of the first layer can be expressed as follows:

(15) $$\begin{array}{c} \Gamma =[\left\{\theta_{ij}\right\},\left\{\gamma_{ij}\right\},\left\{\zeta_{ij}\right\}]\;\;\;\;i\in N,j\in N. \end{array}$$

By efficiently merging two lines into a new one, the second layer constraints can be found:

(16) $$\begin{array}{c} {Ł}_{i}^{\left(2\right)}=\left[p_{i}^{\left(2\right)},l_{i}^{\left(2\right)},k_{i}^{\left(2\right)}\right]\;\;\;\;i\in N-1, \end{array}$$

(17) $$\begin{array}{c} p_{i}^{\left(2\right)}=midpoint\left(p_{i},p_{i+1}\right), \end{array}$$

(18) $$\begin{array}{c} l_{i}^{\left(2\right)}=distance\left(p_{i},p_{i+1}\right), \end{array}$$

(19) $$\begin{array}{c} k_{i}^{\left(2\right)}=slope\left(p_{i},p_{i+1}\right), \end{array}$$

where midpoint and slope are the functions for middle point and slope, respectively. Similarly, the second geometric constraints $\Gamma^{\left(2\right)}$ can be described as follows:

(20) $$\begin{array}{c} \Gamma^{\left(2\right)}=\left[\left\{\theta_{ij}^{\left(2\right)}\right\},\left\{\gamma_{ij}^{\left(2\right)}\right\},\left\{\zeta_{ij}^{\left(2\right)}\right\}\right]\;\;i\in N-1,j\in N-1. \end{array}$$

For a shape-based template consisting of *N* lines, there are *N* layers of geometric constraints that can be efficiently found as follows:

(21) $$\begin{array}{c} \Lambda =\left[\begin{array}{c} \Gamma^{\left(1\right)}\\ \Gamma^{\left(2\right)}\\ \vdots \\ \Gamma^{\left(N\right)} \end{array}\right]=\left[\begin{array}{c} \left\{\theta_{ij}\right\},\left\{\gamma_{ij}\right\},\left\{\zeta_{ij}\right\}\\ \left\{\theta_{ij}^{\left(2\right)}\right\},\left\{\gamma_{ij}^{\left(2\right)}\right\},\left\{\zeta_{ij}^{\left(2\right)}\right\}\\ \vdots \\ \left\{\theta_{ij}^{\left(N\right)}\right\},\left\{\gamma_{ij}^{\left(N\right)}\right\},\left\{\zeta_{ij}^{\left(N\right)}\right\} \end{array}\right]. \end{array}$$

For an image, supposing that there are *n* lines in the 3D matrix of grids, it can be expressed as follows:

(22) $$\begin{array}{cc} \Psi_{i} & =[p_{i},l_{i},k_{i}]\;\;\;\;i\in n. \end{array}$$

For each geometric constraint in Γ, a corresponding candidate set of η pairs of lines can be found. In this candidate set, each pair of lines are efficiently merged into a new line in $\Psi^{\left(2\right)}$. Similarly, for a shaped-based template consisting of *N* lines, it is efficient to extract Ψ for each layer of constraints as follows:

(23) $$\begin{array}{c} \Psi \overset{\Gamma^{\left(1\right)}}{\to }\Psi^{\left(2\right)}\overset{\Gamma^{\left(2\right)}}{\to }\Psi^{\left(3\right)}\overset{\Gamma^{\left(3\right)}}{\to }\ldots .\overset{\Gamma^{\left(N-1\right)}}{\to }\Psi^{\left(N\right)}. \end{array}$$

In the highest layer $\Psi^{\left(N\right)}$, there must be η lines, each of which represents a combination of lines that might satisfy each geometric constraint in $\Gamma^{\left(1\right)}$. By efficiently merging pair lines which satisfy the geometric constraint, it is possible to efficiently detect target objects that mostly satisfy the geometric constraints of the given shape-based template.

**Figure 5:**
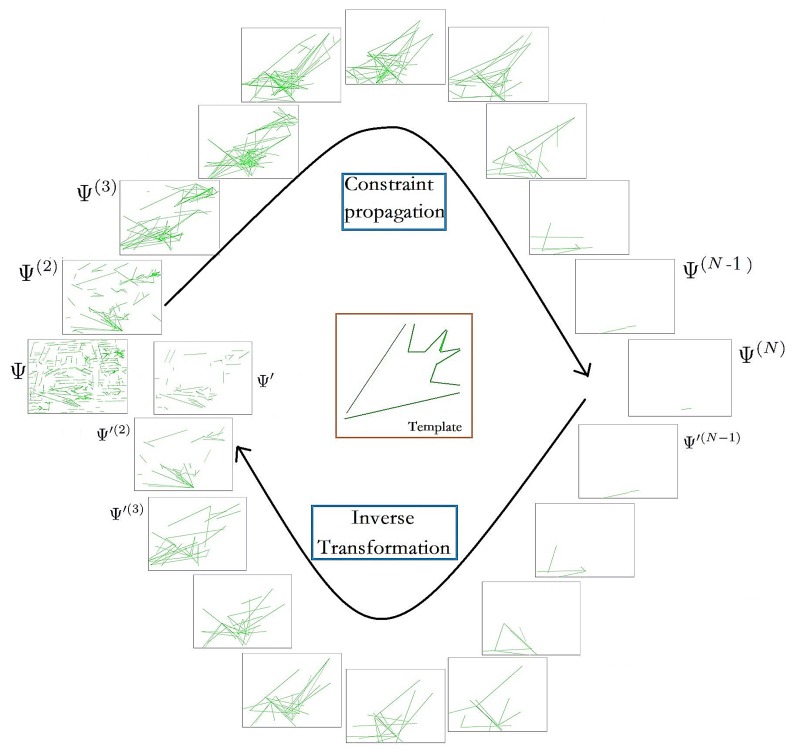
Example of the process of constraint propagation. For a shape-based template consisting of *N* lines, there are *N* layers of constraints $\Gamma^{1}$∼$\Gamma^{N}$. In the first layer, the lines that satisfy the constraints in the first layer are merged into new lines in the second layer. Similarly, the lines that satisfy the constraints are merged into new lines in the next layer. As the layer number increases, the number of lines decreases. Therefore, this indicates that the lines satisfying the constraints can be extracted. For each geometric constraint in $\Gamma^{1}$, a corresponding candidate set of η pairs of lines could be found. In this candidate set, each pair of lines was merged into a new line in $\Psi^{\left(2\right)}$. Similarly, *N* layers of candidate sets of lines could be found as $\Psi^{\left(2\right)}$∼$\Psi^{\left(N\right)}$. In the highest layer $\Psi^{\left(N\right)}$, there must be η lines, each of which represents a combination of lines that might satisfy each geometric constraint in $\Gamma^{\left(1\right)}$. As shown in the figure, in $\Psi^{\left(N\right)}$, one picked line that mostly satisfies the constraint can be inversely transformed into $\Psi^{\prime }$. Through constraint propagation for line-extraction, it is possible to efficiently determine target objects satisfying the shape-based geometric constraints.

As shown in Figure 5, one picked line that mostly satisfies the constraint in $\Psi^{\left(N\right)}$ can be inversely transformed as follows:

(24) $$\begin{array}{c} \Psi^{\prime }\leftarrow \Psi^{\prime (2)}\leftarrow \Psi^{\prime (3)}\leftarrow \ldots \leftarrow \Psi^{\prime (N-1)}\leftarrow \Psi^{\prime (N)}. \end{array}$$

**Figure 6:**
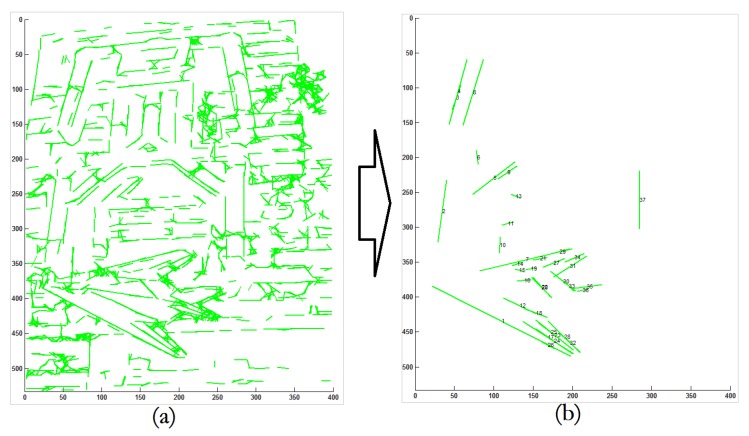
Example of line extraction by constraint propagation. For the image in (**a**), it is efficient to detect the the object satisfying the shape-based geometric constraints, as shown in (**b**).

As shown in the example in Figure 6, it is efficient to detect lines that satisfy the geometric constraints of the shape-based template.

### 4.3. Verification for Object Estimation

**Figure 7:**
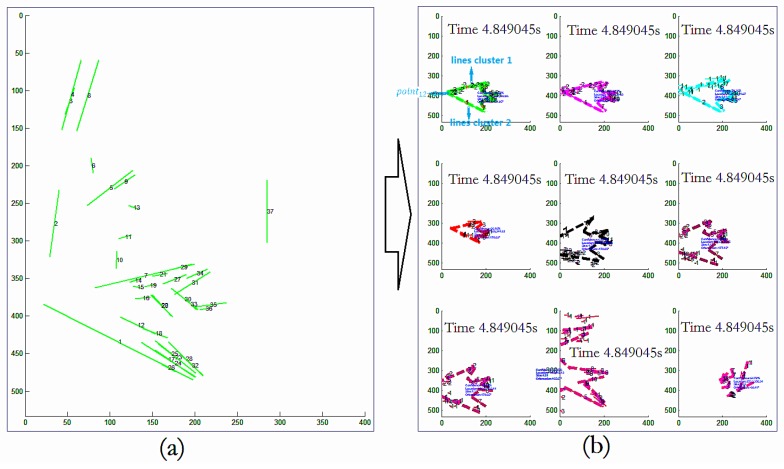
An example for object estimation by verification. For lines in (**a**), the location, scale, and orientation of the object could be verified through searching path. (**b**) The top nine paths satisfying the geometric constraints of Γ were found. Each path represents an estimation of the object. It is obvious that our method not only efficiently detected the object but also estimated the object’s location, scale, and orientation.

The proposed architecture and constraint propagation efficiently detects objects. Then, the object outline can be seen as a path. In the path verification, lines satisfying the geometric constraints of Γ can be found. As in the example shown in Figure 7, it is possible to find the top nine path groups that satisfy the geometric constraints of Γ. The equations can be found as follows:

(25) $$\begin{array}{c} loaction=[min\left(x_{i}\right),min\left(y_{i}\right),max\left(x_{i}\right),max\left(y_{i}\right)],i\in N, \end{array}$$

(26) $$\begin{array}{c} orientation=\left[x^{point_{12}},y^{point_{12}}\right]-\left[\sum_{i=1}^{N}x_{i}/N,\sum_{i=1}^{N}y_{i}/N\right], \end{array}$$

(27) $$\begin{array}{c} size=\frac{norm(\left[x^{point_{12}},y^{point_{12}}\right]-\left[\sum_{i=1}^{N}x_{i}/N,\sum_{i=1}^{N}y_{i}/N\right])}{template}, \end{array}$$

where *N* is the number of lines extracted (e.g., the green lines in Figure 7b, top-left). orientation can be seen as a vector. $point_{12}$ means the cross point of lines cluster 1 and lines cluster 2 (labeled 1 and 2 in Figure 7b, top left, respectively). template means the corresponding length in the template. Each path represents an estimation of the object. The precision of the final estimates of the position, orientation, and size of the found object could achieve 90%. It is obvious that our method not only efficiently detected the object but also estimated its location, scale, and orientation.

## 5. Experimental Results

**Figure 8:**
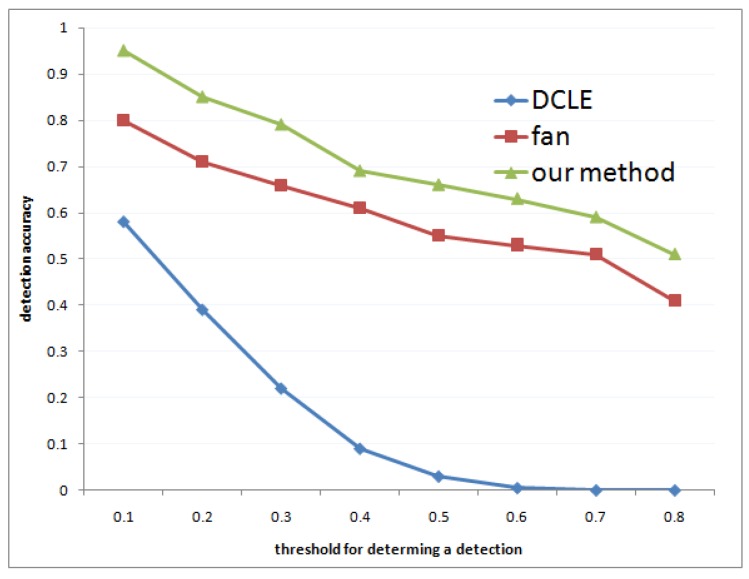
Accuracy of the methods. The horizontal axis shows the threshold for determining a detection, and the vertical axis shows the detection accuracy.

**Figure 9:**
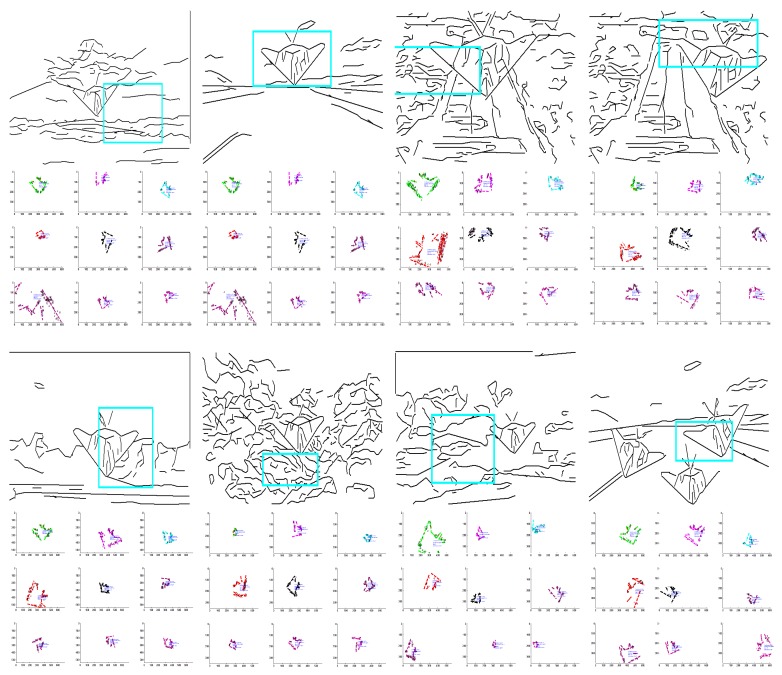
Experimental comparison between our method and DCLE [37,38]. First and third row: detection by DCLE. Second and fourth row: detection by our method. Our method could not only detect the object but could also verify its location, scale, and orientation.

Experimental comparisons were performed on a dataset [36]. The dataset includes 120 images of various resolution. There are flights of various position, scale, and orientation. The dataset is available online [36]. The accuracy of the methods are shown in Figure 8. Figure 9 provides a comparison to DCLE [37,38]. The DCLE method adopts lines, ellipse curves, and SIFT, and when the image backgrounds became complicated, it failed to detect the object. However, our method could cope with complicated images without SIFT and efficiently detected the object by the proposed architecture.

**Figure 10:**
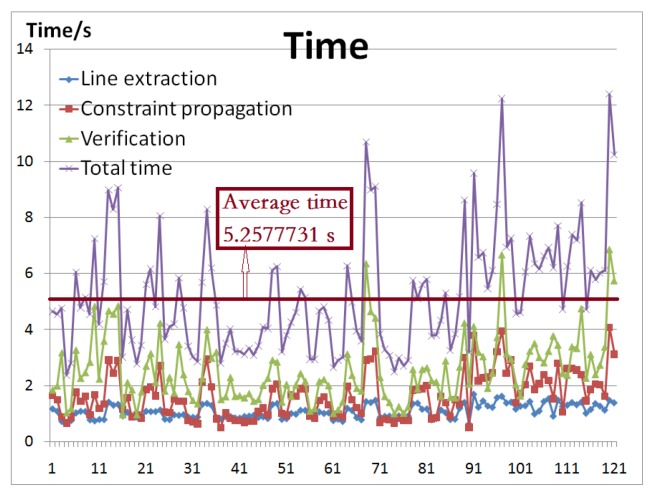
The time our method required for the dataset of images. The horizontal axis shows the input image index, and the vertical axis shows the time the current approach required. For one input image, the total time our method required was approximately 5 s.

**Figure 11:**
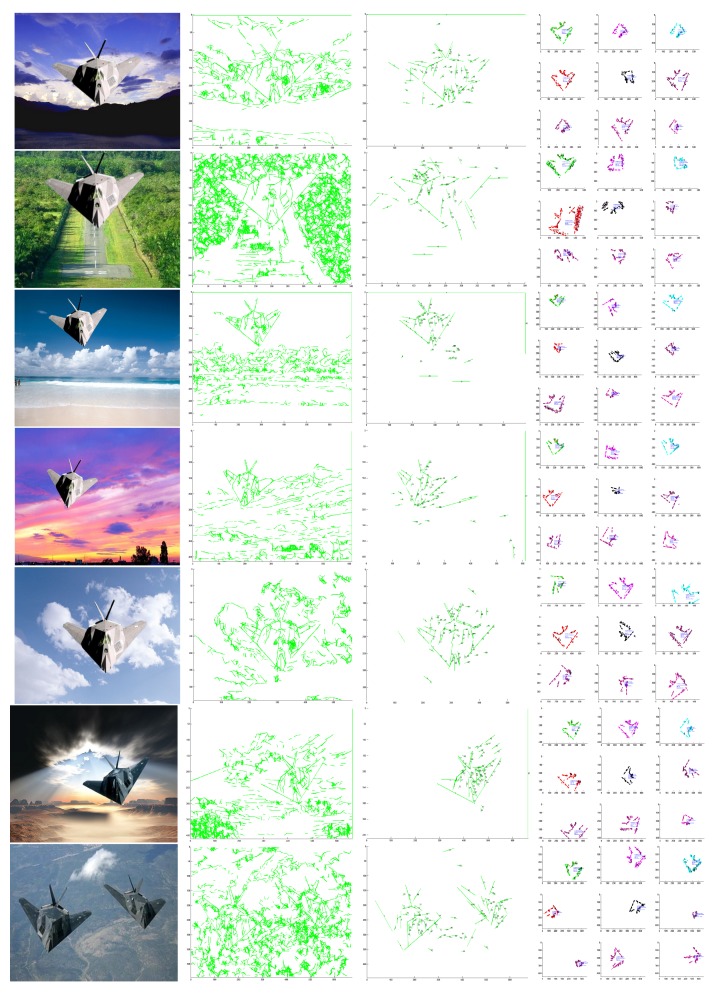
More experiments were performed on a dataset of images with various complicated backgrounds. The first column shows the original image, and the detected lines based on the proposed architecture are shown in the second column. Then, through the efficient approach of constraint propagation, the third column exhibits the detected the object satisfying the geometric constraints of the shape-based template. Finally, the location, scale, and orientation of object could be estimated via the verification shown in the fourth column, including several optimal combinations of lines.

More experiments were performed on a dataset of images with various complicated environments. The time is shown in Figure 10 and examples are shown in Figure 11. The dataset [36] includes 120 images of various resolution, with flights of various position, scale, and orientation. The final results prove that the detection performance was good.

We tested our algorithm on an Intel i5 PC with 8 GB RAM, and the programming language was Matlab. For each image in the dataset [36], the time this method required was quite small. The time taken by the DCLE technique [37,38] and the fan model [23] was around 150 s. With the proposed architecture, it was possible to indicate that the simulated process of line extraction required around 1 s. Through the constraint propagation, the step to efficiently detect the object required around 1.5 s. It took approximately 2 s to verify the information of the detected object (e.g., location, scale, and orientation). For one input image, the total time that our method required was around 5 s, proving that the implementation of the proposed technique allows rapid application to recognize an object-based shape-model, meeting the real-time requirements in robots.

## 6. Conclusions

In this paper, an efficient visual cortex-inspired imaging sensor architecture and an approach of constraint propagation examination are presented to extract and process information (linear contour) from an input image with various complicated environments. Through the proposed imaging sensor architecture and constraint propagation, sensor information could be efficiently processed. The detected lines were stored in a 3D matrix of grids, including the line’s center position, slope, and length. In order to efficiently detect the object, a constraint propagation approach was designed to detect the target object satisfying the geometric constraints of the given shape-based template. Through the verification, the location, scale, and orientation of object could be estimated and reconstructed. The experimental results showed that the implementation of the proposed technique allowed rapid application to efficiently detect the object in a quite complicated environment. In applying this architecture in the preprocessing phase to extract lines, the running time of object detection was decreased dramatically, not only because all lines are represented as very simple vectors, but also because the number of lines is very limited. Without any high-dimensional feature descriptor, long-term training, and time-expensive preprocessing, it takes less time to implement the proposed imaging sensor architecture and constraint propagation approach. The final results showing good performance of the proposed method prove that sensor information can be efficiently processed by the proposed imaging sensor architecture with constraint propagation as the neural architecture.
